# The application effect of rubber band traction assisted endoscopic submucosal dissection for esophageal precancerous lesions

**DOI:** 10.12669/pjms.41.6.12168

**Published:** 2025-06

**Authors:** Wenjuan Zhang, Shi Ding, Xuerong Zhao, Changjuan Li, Haitao Liu, Lidong Ma

**Affiliations:** 1 Wenjuan Zhang, Department of Gastroenterology, Handan First Hospital, Handan, Hebei Province 056000, P.R. China; 2 Shi Ding, Department of Pharmacology, Chengde Medical University, Chengde, Hebei Province 067000, P.R. China; 3 Xuerong Zhao, Department of Immunology, Chengde Medical University, Chengde, Hebei Province 067000, P.R. China; 4 Changjuan Li, Department of Gastroenterology, Handan First Hospital, Handan, Hebei Province 056000, P.R. China; 5 Haitao Liu, Department of Gastroenterology, Handan First Hospital, Handan, Hebei Province 056000, P.R. China; 6 Lidong Ma, Department of Gastroenterology, Handan First Hospital, Handan, Hebei Province 056000, P.R. China

**Keywords:** Endoscopic submucosal dissection, Esophageal precancerous lesions, Rubber band, traction

## Abstract

**Objective:**

This study aimed to evaluate the efficacy of rubber band traction-assisted ESD (RBT-ESD) in treating esophageal precancerous lesions (EPLs).

**Methods:**

This retrospective cohort study included the clinical data of 110 patients with EPLs who received ESD from June 1, 2022 to November 10, 2024 at the Handan First Hospital. Of them, 55 patients who underwent RBT-ESD were matched with patients who underwent conventional ESD (C-ESD) using propensity score matching in a 1:1 ratio. Surgical outcomes, one-time complete resection rate, quality of life (QOL), and incidence of complications were compared between the two groups.

**Results:**

RBT-ESD was associated with considerably lower surgical time, intraoperative mucosal injection volume, submucosal dissection time, mucosal dissection speed, and postoperative hospital stay than C-ESD (*P*<0.05). The one-time complete resection rate of the RBT-ESD group (100%) was higher than that of the C-ESD group (94.5%), but the difference was not significant (*P*>0.05). Post-surgery QLQ-C30 scores in both groups increased and were significantly higher in the RBT-ESD group compared to the C-ESD group (*P*<0.05). The incidence of adverse events was comparable in the two groups (*P*>0.05).

**Conclusions:**

Compared with C-ESD, RBT-ESD for EPLs may optimize the effectiveness of the surgical treatment and enhance patients’ QOL with comparable safety.

## INTRODUCTION

Esophageal cancer (EC) is the ninth most common cancer and the sixth leading cause of cancer deaths worldwide.^1^,^2^ Studies show that early and effective treatment, such as surgery, in patients with esophageal precancerous lesions (EPLs) can significantly improve survival rates.^3^,^4^ With the development of endoscopic technology, endoscopic submucosal dissection (ESD) has been applied in early EC and EPLs.^5^,^6^ ESD has been widely recognized for its advantages of minimal trauma, fewer complications, and faster postoperative recovery.^6^,^7^ However, during conventional ESD (C-ESD), fully exposing the submucosal layer of the lesion can be challenging, which increases the difficulty of the surgery and the risk of perforation and bleeding.^6^-^8^ Various traction assistance devices that have been developed to overcome this difficulty include rubber band clamp traction, the clamp and trap method, and the magnetic traction method.^9^-^11^ Traction can better pull apart the mucosal layer, quickly locate the lesion site, and reduce the risk of adverse events.^10^,^11^

Previous studies have shown that traction-assisted strategies shorten ESD surgery time and reduce the occurrence of adverse events.^9^-^12^ However, the relative advantages of traction-assisted ESD treatment for EC are still unclear. Ota et al.^13^ confirmed that ESD with clip and rubber band assistance is safer and more effective in treating colorectal lesions than traditional ESD. However, Xia et al.^14^ found that traction-assisted ESD is more effective in reducing the average surgical time for EC, but does not impact the incidence of whole block resection or bleeding. This study aimed to compare the outcomes of C-ESD and rummer band traction-assisted ESD (RBT-ESD) in treating EPLs and to evaluate the effectiveness and safety of traction methods in treating EPLs.

## METHODS

Clinical records of patients with EPLs who received ESD treatment at the Handan First Hospital from June 1, 2022 to November 10, 2024 were retrospectively selected.

### Ethical approval:

The Handan First Hospital ethics committee approved this study (approval number: HDYY-LW-25029).

### Inclusion criteria:

- Age>18 years old.
- EPLs confirmed by pathology.^11^
- Receive surgical treatment for RBT-ESD or C-ESD.
- Patients with complete clinical data and follow-up for more than three months.

### Exclusion criteria:

- Patients with organic lesions in kidney and liver.
- Patients with mental disorders.
- Patients with coagulation dysfunction (international standardized ratio>2.0, platelet count<100000/mm3).
- Previous history of endoscopic resection surgery with incomplete resection.
- Pregnancy or breastfeeding.

### The ESD procedure:

A single-channel endoscope (GIF-Q260J; Olympus, Japan), a transparent light shield (D-21-11804; Olympus Co., Japan), an electrosurgical generator (ICC200; ERBE, Germany), and an injection needle (IND2-25423230; Micro-Tech, China) were used. A double blade (MK-T-1-195; Micro-Tech, China) was used as a cutting device. Hot biopsy forceps (HBF-23/2300, Micro-Tech, China). Titanium clip (ROCC-D-26-195; Micro-Tech, China) were used to close wounds and secure rubber bands.

A double knife marking treatment was performed 3mm around the lesion, with one point marked at an interval of 2-mm. Subcutaneously, 100 ml of physiological saline, 1-ml of adrenaline, and 1-ml of methylene blue were injected at the marked points (2ml at each point). Double knives were used to cut open the mucosa (3 mm) at the marked point and peel off the underlying layer of the lesion mucosa.

### C-ESD group:

The mucosa was gradually separated completely from the muscle layer, and the lesion was completely removed in one go.

### RBT-ESD group:

*-Partial submucosal dissection:* A double knife was used to dissect the submucosal layer on the oral side.
*-Rubber band traction:* The rubber band was fixed on the side of the excised lesion to provide traction.
*-Submucosal dissection:* The submucosal space was exposed by traction, and the lesion was completely dissected along the submucosal layer near the muscle layer. If the traction was weakened or too strong during dissection, the strength of the traction was adjusted by blowing and releasing carbon dioxide.
- A new titanium clip was used to remove the clip fixed to the esophagus.

### Postoperative treatment:

Blood vessels exposed on the wound’s surface were treated with electrocoagulation. The specimens were sent for pathological analysis by experienced pathologists independent of endoscopists.

### Data collection:

The collected indicators included patient demographic characteristics (age, gender, and underlying disease status), disease duration, lesion location, maximum lesion diameter, surgical status (surgical duration, intraoperative mucosal injection volume, submucosal dissection time, mucosal dissection speed, hospitalization time, one-time complete resection rate, complications (including intraoperative bleeding, esophageal reflux, recurrence), preoperative and postoperative QOL.

The surgical time was defined as the time between the initial mucosal incision and the completion of resection. The submucosal dissection time was defined as the time required from the start of mucosal incision to complete removal of the diseased tissue. Mucosal dissection speed: resection area (mm^2^)/surgical time (minute).

The length of hospital stay was defined as the cumulative number of days from admission to discharge. The whole block resection was defined as the complete removal of the lesion. Intraoperative bleeding was defined as bleeding that requires endoscopic intervention during ESD. Esophageal reflux was defined as abnormal reflux of gastric contents (including stomach acid and food) into the esophagus, causing irritation and damage to the esophageal mucosa due to stomach acid. The QOL was evaluated based on the Quality-of-Life Questionnaire Core 30 (QLQ-C30). The QLQ-C30 scale has 30 items, with the last two items scoring 1-7 points and all other items scoring 1-4 points, resulting in a total score of 126, with higher scores indicating better QOL.

### Statistical analysis:

Data were analyzed using SPSS 26.0. The Shapiro-Wilk test was employed to evaluate the normal distribution of continuous data. Continuous data were expressed as mean ± standard deviation (SD) or median and interquartile range (depending on the situation). Categorical variables were represented as numbers and percentages. For continuous data, statistical significance is calculated using the student t-test or Mann Whitney U test, which includes age, disease duration, and length of hospital stay. The classification data uses Pearson’s chi-square test or Fisher’s exact test, such as gender, lesion location, and intraoperative bleeding. *P*<0.05 indicated a statistically significant difference.

## RESULTS

This study included 110 patients with EPLs who underwent ESD from June 1, 2022 to November 10, 2024. Patient ages ranged from 45 to 78 years, with an average of 63.3 ± 6.6 years. The cohort consisted of 61 males and 49 females. A total of 55 patients who underwent RBT-ESD were matched with the cohort of C-ESD patients using propensity score matching in a 1:1 ratio. There were 29 males (52.7) and 26 females (47.3) in the RBT-ESD group, with age range of 45-76 years, and an average age of 62.4 ± 7.1 years. The C-ESD group consisted of 32 males and 23 females, aged 53-78 years, with an average age of 64.3 years. There was no significant difference between the two groups in age, sex, course of disease, location of lesion, maximum diameter of lesion, diabetes, hyperlipidemia, hypertension, and other baseline characteristics (*P*>0.05) (Table-I).

**Table-I:** Comparison of baseline characteristics between two groups.

Characteristics		RBT-ESD group (n=55)	C-ESD group (n=55)	t/χ^2^	P
Age (years), mean±SD		62.4±7.1	64.3±6.0	-1.532	0.128
Sex, n(%)	Male	29 (52.7)	32 (58.2)	0.213	0.644
	Female	26 (47.3)	23 (41.8)	
Disease duration (years), mean±SD		2.37±1.15	2.53±1.07	0.751	0.454
The location of the lesion, n(%)	Upper esophagus	16 (29.1)	22 (40.0)	1.461	0.482
	Middle esophagus	30 (54.5)	25 (45.5)		
	Lower esophagus	9 (16.4)	8 (14.5)	
Maximum diameter of lesion (cm), mean±SD		1.87±0.43	1.93±0.51	-0.586	0.559
Diabetes (yes), n(%)		10 (18.2)	6 (10.9)	1.170	0.279
Hyperlipidemia (yes), n(%)		13 (23.6)	9 (16.4)	0.909	0.340
Hypertension (yes), n(%)		15 (27.3)	16 (29.1)	0.045	0.832

***Note:*** RBT-ESD, rubber band traction assisted endoscopic submucosal dissection; C-ESD, conventional endoscopic submucosal dissection.

As shown in Table-II, the surgery time, the submucosal dissection time, the mucosal dissection speed, and the postoperative hospitalization time were significantly lower in the RBT-ESD group than in the C-ESD group (*P*<0.05). The one-time complete resection rate (100%) of the RBT-ESD group was somewhat higher than that of the C-ESD group (94.5%), but the difference was not statistically significant (*P*>0.05). In the RBT-ESD group, there were eight incidences (14.5%) of adverse events, including five cases of intraoperative bleeding, two cases of esophageal reflux, and one case of recurrence. Ten incidences of adverse events in the C-ESD group (18.2%) included six cases of intraoperative bleeding, two cases of esophageal reflux, and two cases of recurrence with no significant difference in the incidence of various adverse events between the two groups (all *P*>0.05) (Table-II).

**Table-II:** Comparison of surgical outcomes between two groups.

Variables		RBT-ESD group (n=55)	C-ESD group (n=55)	t/χ^2^	P
Surgery time, min		41.1±5.9	55.6±14.5	-6.888	<0.001
Intraoperative mucosal injection volume, ml		26.7±4.7	38.9±8.6	-9.208	<0.001
Submucosal dissection time, min		27.2±5.4	44.3±10.7	-10.524	<0.001
Mucosal dissection speed, mm^2^/min		37±10	24±9	0.266	<0.001
Hospitalization time, day		4 (3-5)	4 (3-6)	-2.680	0.007
One-time complete resection rate, n(%)	Yes	55 (100)	52 (94.5)	3.084	0.242
	No	0 (0)	3 (6.5)		
Adverse event, n(%)	Yes	8 (14.5)	10 (18.2)	0.067	0.797
	No	47 (85.5)	45 (81.8)		
Intraoperative bleeding, n(%)	Yes	5 (9.1)	6 (10.9)	0.101	0.751
	No	50 (90.9)	49 (89.1)		
Esophageal reflux, n(%)	Yes	2 (3.6)	2 (3.6)	0.000	1.000^b^
	No	53 (96.4)	53 (96.4)		
Recurrence, n(%)	Yes	1 (1.8)	2 (3.6)	0.343	1.000^b^
	No	54 (98.2)	53 (96.4)		

***Note:*** b, Continuity correction. RBT-ESD, rubber band traction assisted endoscopic submucosal dissection;

C-ESD, conventional endoscopic submucosal dissection.

**Fig.1:**
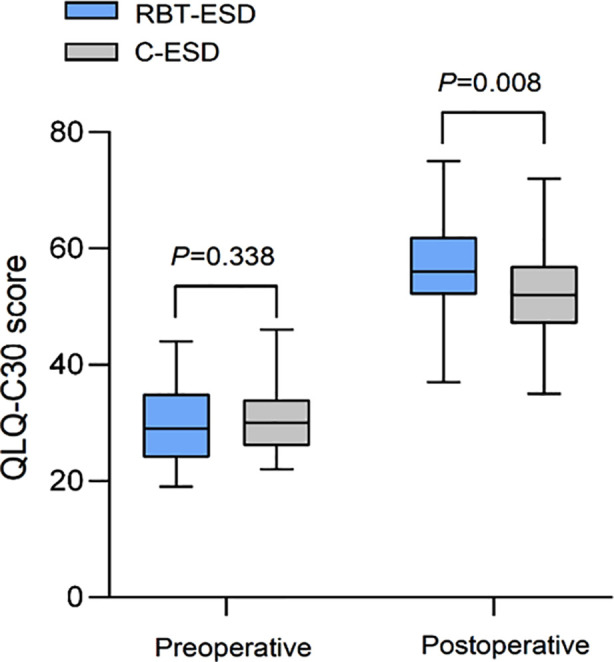
Comparison of QLQ-C30 scores between two groups.

Preoperative QLQ-C30 scores were comparable in the two groups. After the surgery, the QLQ-C30 scores of both groups increased compared to preoperative levels and were considerably higher in the RBT-ESD group than the C-ESD group (*P*<0.05) (Fig.1).

RBT-ESD, rubber band traction assisted endoscopic submucosal dissection; C-ESD, conventional endoscopic submucosal dissection; QLQ-C30, Quality of Life Questionnaire Core 30.

## DISCUSSION

This retrospective study compared the efficacy and safety of RBT-ESD and C-ESD in treating patients with EPLs. The results indicate that RBT-ESD is superior to C-ESD in terms of surgery time, intraoperative mucosal injection volume, submucosal dissection time, mucosal dissection speed, hospitalization time, and postoperative QOL. There was no significant difference in the incidence of adverse events such as one-time complete resection rate, intraoperative bleeding, esophageal reflux, and recurrence between the two groups.

EC is one of the most common malignant tumors in China.^1^,^15^ Early detection, diagnosis, and treatment are key to improving the survival rate of EC patients.^2^,^3^,^15^,^16^ ESD is considered a method of choice for treating early EC and EPLs due to the low invasiveness of the procedure.^6^,^7^,^17^ It can significantly improve the overall resection rate of large-sized or irregularly shaped lesions without affecting the QOL of patients.^17^,^18^

However, ESD still poses certain challenges as it is associated with longer surgical times, which may increase the risk of adverse events.^17^-^19^ Traction, also known as the ’third hand’ of endoscopic treatment, quickly and effectively pulls apart the mucosal layer, thereby improving the speed of submucosal dissection, shortening the surgical time, and lowering the risk of complications.^20^,^21^ Ota et al.^22^ showed that clip traction shortened surgical time and was associated with higher safety in patients with esophageal ESD. A study by Xie et al.^23^ confirmed that traction can reduce the incidence of intrinsic muscle layer injury and shorten the dissection time, which is in agreement with the results of this study and further confirms that ESD is a safe and effective adjuvant surgery for treating esophageal ESD.

The results of this study also indicate that traction-assisted technology can significantly improve the operational efficiency of ESD surgery. Traction-assisted ESD can better expose the surgical field, allowing the operator to perform submucosal dissection quickly and reducing unnecessary surgery time and mucosal replenishment injection volume. Studies have shown that longer surgical time is a risk factor for endoscopic perforation and bleeding.^24^,^25^ Similarly, improving surgical speed may reduce the risk of complications and alleviating the fatigue of endoscopists. However, this study found no significant difference between the two groups in terms of one-time complete resection rate and incidence of various adverse events. This discrepancy may be due to the small sample size or the variability in the expertise of the ESD-performing operators.

A study by Dai et al.^26^ found no significant difference in the incidence of complications between patients undergoing traction-assisted ESD surgery and those undergoing C-ESD surgery, which is consistent with the results of this study. In ESD surgery, corresponding hemostatic measures, such as electrocoagulation hemostasis, are performed once bleeding occurs. It is plausible that these measures were equally efficient in preventing complications in the RBT-ESD and the C-ESD groups.

The results of this study indicate that while QLQ-C30 scores in both groups increased after surgery, RBT-ESD was associated with a better quality of life compared to the C-ESD. This suggests that while ESD in general can improve the QOL of patients to a certain extent, traction-assisted ESD procedure has a more significant impact on improving the QOL of patients. Patients with EPLs may experience changes in physical function and psychological state as a result of pathological and procedural factors. Traction-assisted ESD technology comprehensively reduces surgical trauma, shortens surgical time, and lowers the incidence of complications.^23^,^24^ This can help patients recover faster after surgery, improving their QOL.^23^-^25^

This study has adopted a combination of rubber bands and clamping methods. These are simple, economical, convenient, effective, and easy-to-execute techniques without complex equipment and accessories, making them an ideal traction method for esophageal ESD. Firstly, rubber bands are inexpensive and easy to obtain. Traction can reduce surgical time, lower the risk of perforation, and is minimally invasive. Secondly, the internal traction method does not require reinsertion of the endoscope. It uses clips connected by rubber bands, which can pass through the endoscope’s instrument channel.^27^ The system is separate from the endoscope and, therefore, is not limited by the movement of the endoscope. Based on previous studies in colorectal ESD, it has been shown that traction can be adjusted by adjusting the inflation and deflation of the gas.^13^,^27^,^28^ In addition, RBT-ESD is a relatively simple technique that can be easily adopted by endoscopists.^27^,^28^ In summary, RBT-ESD is an effective, safe, and cost-effective traction technique for EPLs.

### Limitations

First, it is a retrospective analysis with some selection bias. Second, as it is conducted in a single center, extensive validation from multiple centers is required. Third, the small sample size may lead to statistical differences in the specimen area. Fourth, the study only compared RBT-ESD with C-ESD and did not compare it with other traction methods. Finally, C-ESD and RBT-ESD surgeries were performed by different teams of endoscopists. Therefore, the variability in their skills may affect the results.

## CONCLUSION

Compared with C-ESD, RBT-ESD has significant advantages in treating patients with EPLs, can improve surgical treatment, enhance patients’ QOL, and does not increase the risk of adverse events. Future studies should compare RBT-ESD with other traction methods and conduct multi-center, prospective trials to provide better medical services for patients with EPLs.

### Authors’ contributions:

**WZ:** Literature search**,** study design and manuscript writing.

**SD, XZ, CL, HL and LM:** Data collection, data analysis and interpretation. Critical Review.

**WZ:** Manuscript revision and validation and is responsible for the integrity of the study.

All authors have read and approved the final manuscript.
